# Draft Genome Sequence of Streptococcus canis Clinical Strain OT1, Isolated from a Dog Owner with Invasive Infection without a Dog Bite in Japan

**DOI:** 10.1128/MRA.00770-19

**Published:** 2019-09-26

**Authors:** Haruno Yoshida, Yukie Katayama, Yasuto Fukushima, Hirofumi Ohtaki, Kiyofumi Ohkusu, Tetsuya Mizutani, Takashi Takahashi

**Affiliations:** a Laboratory of Infectious Diseases, Kitasato Institute for Life Sciences & Graduate School of Infection Control Sciences, Kitasato University, Minato-ku, Tokyo, Japan; b Research and Education Center for Prevention of Global Infectious Diseases of Animals, Tokyo University of Agriculture and Technology, Fuchu, Tokyo, Japan; c Department of Medical Technology, Kansai University of Health Sciences, Kumatori, Osaka, Japan; d Department of Microbiology, Tokyo Medical University, Shinjuku-ku, Tokyo, Japan; University of Maryland School of Medicine

## Abstract

Streptococcus canis is a β-hemolytic bacterium that can cause invasive infections in animals and humans. Here, we report a draft genome sequence of S. canis strain OT1, isolated from a female dog owner with bacteremia without a dog bite. The draft genome comprises 2,030,366 bp in 48 contigs.

## ANNOUNCEMENT

Streptococcus canis, which was first reported in 1986 (1), is a β-hemolytic bacterium that can cause mild or severe infections in animals and humans (2–4). We previously reported a draft genome sequence of S. canis strain TA4 with the *scm* gene encoding the M-like protein (5) that was isolated from a male dog owner with bacteremia caused by a dog bite (6). A case of bacteremia due to S. canis strain OT1 in a female dog owner was described, and it was the first report of human bacteremia caused by S. canis without a dog bite in Japan (7). The owner kept a dog in her room and often slept with it on the same bed. Here, we report a draft genome sequence for this isolate (strain OT1).

S. canis strain OT1 (7) was grown in Todd-Hewitt broth supplemented with yeast extract overnight. Genomic DNA was extracted using the DNeasy blood and tissue kit (Qiagen) after pretreatment with lysozyme and proteinase K. A DNA sequencing library was prepared using the Nextera XT DNA sample prep kit (Illumina). The library was indexed and sequenced using an Illumina MiSeq benchtop sequencer.

Sequencing yielded 3,742,902 reads (876,346,161 bp), and the reads were trimmed with the quality trimming tool of the CLC Genomics Workbench (ver. 6.5.1), with default parameters. The *de novo* assembly was performed using the CLC Genomics Workbench with a modified parameter, in which the minimum contig length setting was changed from 200 bp to 500 bp. The assembled genome consisted of 48 contigs (GenBank accession number BJOW01000000) ranging in size from 682 to 243,926 bp, with an average coverage of 427× and an *N*_50_ value of 137,776 bp. The draft genome sequence was automatically annotated using the DDBJ Fast Annotation and Submission Tool (DFAST; https://dfast.nig.ac.jp/) (8). The length of the draft genome was 2,030,366 bp (39.7% GC content), and it contained 1,931 coding sequences (CDSs), 38 tRNAs, and 3 rRNAs, indicating a coding ratio of 84.5%. It also included one incomplete phage element consisting of 31 proteins and a type I-C CRISPR-Cas system (contig 3).

Comparative genome analysis using the mapping procedure was conducted by using the mapped reads to reference tool with default parameters that could be originally used in the CLC Genomics Workbench (ver. 6.5.1). This procedure revealed that 91.16% of the reads from the OT1 sequence were also present in the complete genome sequence of S. canis type strain NCTC12191 (GenBank accession number LR134293) of bovine origin. We attempted *de novo* assembly using the remaining 330,743 reads, which yielded 29 contigs with 163 CDSs in 157,907 bp. The unmapped reads included an incomplete phage element (8,916 bp) with 15 proteins that was located in contig 53 and possessed CDSs that differed from those of the incomplete phage element in contig 3, suggesting that the original element was not present in strain NCTC12191. Many of the CDSs in these contigs encoded phage-derived proteins with similarity to phage and phage-associated proteins shared among pathogenic streptococci. The matched reads contained sequences encoding several virulence factors, such as proteases, toxins, hemolysins, adhesins, and others, that were identical to those in NCTC12191, using the VFDB (http://www.mgc.ac.cn/VFs/main.htm).

### Data availability.

The draft genome sequence of strain OT1 has been deposited in DDBJ/EMBL/GenBank under the accession number BJOW01000000. The raw read data can be accessed in the DDBJ SRA under accession number DRA008579.
